# How to Proceed with Asymptomatic Modular Dual Taper Hip Stems in the Case of Acetabular Revision

**DOI:** 10.3390/ma13051098

**Published:** 2020-03-02

**Authors:** Thomas M. Grupp, Marc Baxmann, Volkmar Jansson, Henning Windhagen, Karl-Dieter Heller, Michael M. Morlock, Hanns-Peter Knaebel

**Affiliations:** 1 Aesculap AG Research & Development, 78532 Tuttlingen, Germany; marc.baxmann@gmx.de; 2 Department of Orthopaedic Surgery, Physical Medicine & Rehabilitation, Campus Grosshadern Ludwig Maximilians University Munich, 81377 Munich, Germany; volkmar.jansson@med.uni-muenchen.de; 3 Department of Orthopaedic Surgery, Hannover Medical School, 30625 Hannover, Germany; henning.windhagen@diakovere.de; 4 Department of Orthopaedic Surgery, Herzogin-Elisabeth-Hospital, 38124 Braunschweig, Germany; kd.heller@heh-bs.de; 5 Institute of Biomechanics, TUHH Hamburg University of Technology, 21073 Hamburg, Germany; morlock@tuhh.de; 6 Roechling SE & Co. KG, 68165 Mannheim, Germany; hp.knaebel@roechling.com

**Keywords:** total hip arthroplasty, dual taper modular hip stem, acetabular revision, asymptomatic stem modularity, decision making model, threshold

## Abstract

How to proceed with a clinically asymptomatic modular Metha® Ti alloy stem with dual taper CoCr neck adapter in case of acetabular revision? To systematically answer this question the status of research and appropriate diagnostic methods in context to clinically symptomatic and asymptomatic dual taper stem-neck couplings has been evaluated based on a systematic literature review. A retrieval analysis of thirteen Metha® modular dual taper CoCr/Ti alloy hip stems has been performed and a rational decision making model as basis for a clinical recommendation was developed. From our observations we propose that in cases of acetabular revision, that for patients with a serum cobalt level of > 4 µg/L and a Co/Cr ratio > 3.6, the revision of the modular dual taper stem may be considered. Prior to acetabular revision surgery a systematic diagnostic evaluation should be executed, using specific tests such as serum metal (Co, Cr) ion analysis, plain antero-posterior and lateral radiographs and cross-sectional imaging modalities (Metal Artefact Reduction Sequence Magnetic Resonance Imaging). For an asymptomatic Metha® dual taper Ti alloy/CoCr stem-neck coupling at the stage of acetabular revision careful clinical decision making according to the proposed model should be followed and overreliance on any single examination should be avoided, considering the complete individual differential diagnosis and patient situation.

## 1. Introduction

Failure of total hip arthroplasty (THA) is a relatively rare condition and the most common causes for revision are aseptic loosening, dislocation, septic loosening and peri-prosthetic fractures [1,2]. For a better adaptation to different diaphyseal and extra-diaphyseal anatomical conditions a modular dual taper neck design was clinically introduced for primary THA by Toni et al. [3] in 1995. Using an anatomic cementless stem design in combination with a modular rectangular tapered neck adapter in a consecutive series of 216 hip arthroplasties they reported a survival rate of 98.6% at 5 years including all implant related complications [3]. Clinical benefits of modular dual taper neck adapter hip stems are the restoration of the hip centre of rotation in combination with an adequate soft tissue balancing in complex anatomical, muscular and ligamenteous patient situations [4,5,6]. For anatomic differences between specific patients (e.g., dysplasia), as well as different hip morphologies in males and females in regard to femur size, Caput-Collum-Diaphysis (CCD) angle, femoral offset and anteversion [7], these designs allow to adjust the native femoral anteversion and offset to restore an adequate abductor muscles lever arm [5], limb length [7] and patients’ original biomechanics [8], independently of the stem size and femoral fixation [6,9]. In an exploratory study on 684 mono-bloc and 125 THAs with a modular neck in patients dedicated to a rapid recovery programme based on selected cases with primary arthritis in an otherwise anatomically normal hip joint, Gerhardt et al. [10] did not find a clear benefit in restoring hip geometry and dislocation rate—only the abductor moment arm which is closely correlated to the femoral offset was better reconstructed in the modular neck adapter cohort. For this “straightforward“ THA the exclusion criteria were profound acetabular dysplasia, discrepancy in leg length or anatomical deformities of the proximal femur [10].

The mid- to long-term clinical results for some modular dual taper stem designs are comparable to the implant survivorship of mono-bloc cementless stem designs [7,9,11,12,13,14,15]. Examining the effectiveness of neck modularity in THA considering sex differences in hip morphology, Traina et al. [7] retrospectively reviewed the clinical results of 2131 modular stems implanted in 1051 men and 1080 women. They reported an estimated Kaplan-Meier survival rate at 11 years of 97.6% for men and 96.0% for women without any modular Ti neck failures [7]. In a series of demanding THA procedures with developmental dysplasia of the hip in 47 patients with 61 modular neck stems with an average follow-up of 117.2 months (range 57–167) Traina et al. [9] reported a cumulative survival of 97.5% at 11 years (one ceramic liner fracture). Analysing the long-term survivorship and complication rate of a cementless modular neck primary stem design in the data base of the Emilia-Romagna registry of orthopaedic prosthetic implants, Fitch et al. [12] found for 692 modular THAs implanted in 26 orthopaedic units an overall Kaplan-Meier survivorship of 95.8% at 12 years follow-up, similar to the 96.1% for all mono-bloc cementless stem designs implanted during the same period.

A major disadvantage of dual taper modular hip stems is the additional interface at the neck-stem junction, increasing the risk for fatigue fracture, fretting, crevice corrosion and metal particle and ion release [6,16,17,18,19,20,21,22]. For Ti alloy hip stem designs with Ti alloy neck adapters, the main implant-related clinical failure mode is fatigue fracture of the neck taper in the highly stressed neck-stem junction due to fretting corrosion, local stress concentration and crack initiation by multi-directional bending and torsion [16,23,24,25,26,27]. For CoCr neck adapters only few fatigue failures have been reported clinically for a Profemur Z titanium alloy stem (Profemur Hip System, Wright, Arlington, TN, USA) with long 8° varus type CoCr alloy modular necks in overweight or obese patients with considerable physical activity [28,29,30]. For CoCr neck adapters, the main clinical failure mechanism is the generation of particulate Co and Cr wear debris and metal ion release from the neck adapter due to tribo-corrosion, that may cause adverse local tissue reactions (ALTR) [17,31,32,33,34] like osteolysis, metallosis and pseudo-tumor formation in the surrounding tissue [19,35,36] as well as elevated serum ion levels [17,33,37,38,39] and systemic toxicity [40,41]. This failure mode is predominantly occurs in mixed CoCr neck/Ti alloy stem junctions [31,33,39,40,42,43,44], but has also been described for Ti alloy/Ti alloy dual taper stems [37,38] and single alloy CoCr couplings [45,46].

In a previous study [16] clinical fatigue failures for a short modular Ti alloy stem in combination with a Ti alloy neck adapter and the influence of the implant material on the endurance behaviour have been described. The current study is focused on the evaluation of possible clinical failure modes related to the effects of particulate debris and ion release due to mechanically assisted fretting and crevice corrosion from the same stem in combination with a CoCr neck adapter in order to derive a clinical decision making model.

## 2. Objectives

The objectives of our study therefore to attempt to answer the following questions:

1) Which suggestions are given in literature how to proceed with clinically symptomatic and asymptomatic dual taper stem-neck couplings?—Status of research and appropriate diagnostic methods.
2) What are the relevant findings based on neck adapter retrievals of the Metha® dual taper CoCr/Ti alloy hip stem design?
3) How to proceed with a clinically asymptomatic dual taper modular hip stem in case of acetabular revision?—Definition of a rational decision making model as basis for a clinical recommendation.

## 3. Literature on Symptomatic and Asymptomatic Dual Taper Hip Stems

**Scheme 1:**
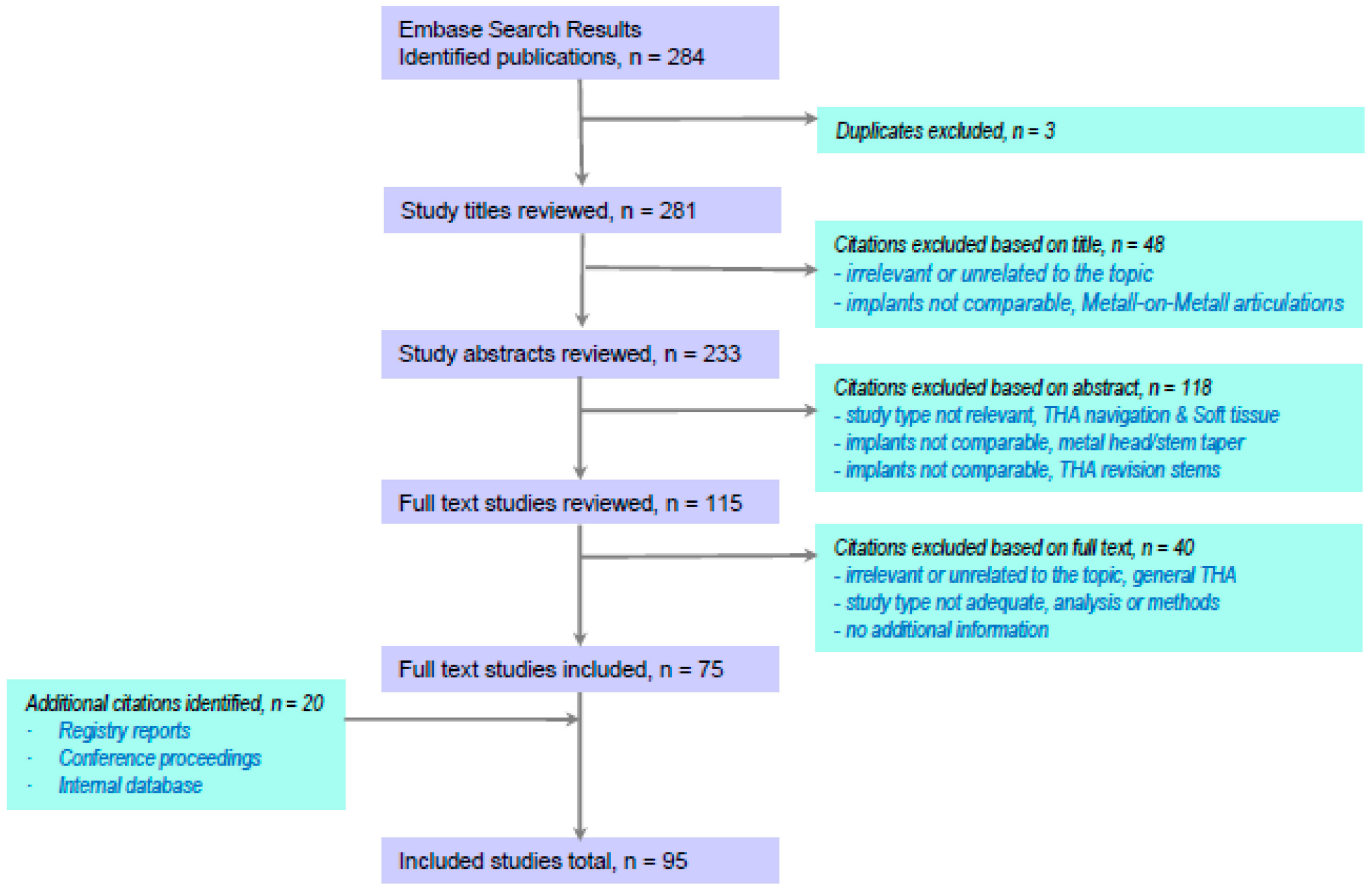
Systematic literature review (Embase n = 284 publications) and studies included in meta-analysis (n = 95).

Searching for total hip arthroplasty with dual taper or bi-modular neck adapters in combination with crevice or tribo-corrosion, particle release, metallosis or adverse local tissue reactions we performed a systematic review in PubMed and EMBASE to present an actual literature overview (time frame 1 January 2006 to 31 January 2020). We found after removal of duplicates n = 281 publications, within n = 166 are not related to the topic of dual taper hip stems. Undergoing a systematic full text review based on n=115 publications we found n = 75 suitable publications and identified additional n = 20 publications (registry reports, conference proceedings, et al.) from our internal database including in total n = 95 studies into the meta-analysis (Scheme 1).

In a review, Mistry et al. [19] postulated etiologies like modular head-neck or neck-stem junctions wear, corrosion damage and metal ion release as “trunnionosis”, a cause of failed total hip arthroplasties [47]. They described the effects of the femoral head size as well as the trunnion design and localized biological reactions associated with trunnionosis [19]. Jacobs et al. [17] described a variety of factors, like head size, taper geometry, material composition, metallurgical processing, surface finish, neck offset and length, design-related factors and a contamination of the taper interface during assembly which may contribute to mechanically assisted crevice corrosion (MACC) in modular junctions involving at least one CoCr component. They found adverse local tissue reactions (ALTR) with clinically symptoms in eleven patients due to MACC with 19-fold elevated mean serum cobalt levels for a beta-titanium alloy (TiMo_12_Zr_6_Fe_2_) modular stem with CoCr neck compared to well-functioning primary THA patients with metal-on-polyethylene articulations at a relatively early post-operative period (mean 7.9 months) [17]. Dimitriou et al. [33] reported about the early outcomes of revision surgery for taper corrosion of two dual taper THA designs (Rejuvenate & ABG II) with CoCr neck and beta-titanium alloy stem based on 198 revision surgeries in 187 patients. They described a significant decline of patients serum ion levels for cobalt from 5.3 µg/L (range 2.3 to 48.5 µg/L) to 1.4 µg/L (range 0.2 to 8.8 µg/L) and for chromium from 2.6 µg/L (range 0.2 to 64 µg/L) to 0.7 µg/L (range 0.1 to 3.9 µg/L) after revision surgery, with a half-life of 3.2 months for cobalt and of 5 months for chromium. The cobalt/chromium ratio also significantly decreased from 4.7 (range 2.1 to 35) to 2.2 (range 0.4 to 8.8) [33].

For the Rejuvenate dual taper modular neck stem consisting of a CoCr neck and a beta-titanium alloy stem, Bernstein et al. [44] described a revision rate of 86% (63 of 73 hip stems) at a mean follow-up of 4.2 years (range 3 to 5.5) with indications for revision surgery being a serum cobalt ion level > 4 µg/L, persistent pain or abnormal MRI (Magnetic Resonance Imaging) findings. They reported mean serum cobalt and chromium ion levels prior to revision of 10.0 µg/L (range 0.3 to 40.0 µg/L) and of 2.3 µg/L (range 1.0 to 7.4 µg/L), respectively and found a substantial decrease of cobalt levels post-operatively. The unrevised group had a serum cobalt level of 2.1 ± 2.0 µg/L and chromium of 1.2 ± 0.4 µg/L, whereas the patients with abnormal MRI findings had 8.1 µg/L (0.3 to 28.9) and 2.0 µg/L (1.0 to 7.0), respectively [44]. Meftah et al. [31] examined the rate of corrosion-related failure and survivorship of the dual taper Rejuvenate stem (n = 97) and correlated implant and patient factors with serum cobalt ion levels and revisions. The Kaplan-Meier survivorship was 40% at four years and the mean cobalt and chromium levels related to metal corrosion in symptomatic patients were 8.1 µg/L (range 0.4 to 31 µg/L) and 2.5 µg/L (range 0.2 to 4.3 µg/L), respectively.

In contrast to that Vundelinckx et al. [39] described for the ABG II dual taper stem design, which was also made of beta-titanium alloy with CoCr neck, only one revision out of a cohort of 306 THAs consisting of a ceramic-on-polyethylene or a ceramic-on-ceramic bearing interface implanted between 2007 and 2011. The patient undergoing a revision developed intermittent pain in the trochanteric area 2 years post-operatively and showed after 4 years peri-prosthetic fluid accumulation and a soft tissue mass around the proximal stem and neck region and an increased serum level of 7.4 µg/L for cobalt. Taking a randomized sample of 19 asymptomatic patients, 9 patients presented a cobalt level > 4 µg/L with a maximum of 7.5 µg/L.

Walsh et al. [48] studied 103 THA revision cases (78 Rejuvenate; 25 ABG II) with dual taper modular neck at a mean time of 2.4 years (range 0.66 to 5) from index surgery to revision and found a mean serum cobalt level of 7.6 µg/L (range 1.1 to 23 µg/L) and a mean serum chromium level of 1.8 µg/L (range 0.1 to 6.8 µg/L). They performed an aspiration of the hip for synovial fluid metal content prior revision THA in 40 patients and found a mean cobalt level of 916 µg/L (range 12 to 3900 µg/L) and a mean chromium level of 599 µg/L (range 3.4 to 3300 µg/L) in the synovial fluid [48].

Barlow et al. [49] reported about 54 patients undergoing revision surgery with 59 revised Rejuvenate dual taper stems based on a cohort of 199 THAs implanted between 2010 and 2012 by a senior surgeon. They analysed the serum ion levels prior to revision and the decline of serum levels at 6 weeks and 12 weeks after revision surgery. For 49 patients with unilateral THA they found a mean serum cobalt ion level of 8.19 ± 5.54 µg/L, which significantly decreased in all patients to 2.68 ± 2.67 µg/L at 6 weeks and to 1.58 ± 1.57 µg/L at 3 months. In five patients with bilateral modular Rejuvenate hip arthroplasty they measured a pre-revision serum cobalt level of 13.33 ± 6.45 µg/L and also a significant drop to 3.73 ± 2.19 µg/L at six weeks post-revision. An overview of additional studies analyzing serum levels of cobalt, chromium and Ti alloy is given for modular dual taper hip designs with TiAl_6_V_4_/TiAl_6_V_4_ and CoCr_29_Mo_6_/TiMo_12_Zr_6_Fe_2_ neck stem couplings [50,51,52,53,54] (Table 1).

In addition, Hussey and McGrory [56] performed a ten years cross-sectional study including 1352 consecutive patients with metal-on-polyethylene THA combined with 12/14 trunnion Ti alloy stem types and found symptomatic MACC present in 43 cases (3.2%). A dual taper Ti alloy neck and Ti alloy stem design (M/L Taper) showed a higher prevalence (4.9%) of MACC than all other Ti alloy stems combined (1.2%) of the same manufacturer. For these stem design (M/L Taper Kinectiv) with a bi-modular Ti alloy neck on a Ti alloy stem and a CoCr 40 mm head, Canham et al. [57] described in a case study a characteristic peri-prosthetic pseudo-tumor formation as an ALTR and an elevated serum cobalt level of 12.3 µg/L and chromium of 1.8 µg/L whereby the only potential source was the head-neck junction.

For symptomatic patients with dual taper hip arthroplasty Kwon et al. [58] propose a differential diagnosis of the temporal onset, duration, severity and location of pain and recommend that also additional symptoms as a swelling or feeling of fullness around the hip should be elicited. To evaluate dual-taper modular implants with corrosion related metal debris contamination, the analysis of serum inflammatory markers as erythrocyte sedimentation rate and C-reactive protein as well as hip aspiration for synovial fluid counts are described to differentiate and exclude periprosthetic joint infection [59]. The elevation of metal ion serum levels and an increase in the cobalt/chromium ratio is also observed [18,40,59].

Adverse local tissue reactions associated with metal ion and debris released by a dual taper Rejuvenate stem were identified in 36 revised stems out of a cohort of 118 THAs by Ghanem et al. [43]. The symptoms of the 36 THAs which were considered as failed began at a mean post-operative time of 14.8 months (range 2.8–34.8) and the average time to revision was 24.1 months (range 8.8–50.2). The authors described higher cobalt serum levels in the failed group of 9.5 ± 6.8 µg/L (range 1.9–24.7) compared to the asymptomatic group of 4.9 ± 3.6 µg/L (range 0.1–15.7) and higher cobalt/chromium ratios of 5.2 ± 3.2 for the failed compared to 3.6 ± 2.3 for the asymptomatic group. However, they reported no correlation in diagnostic accuracy for ALTR, while MRI scan considering pseudo-tumor size was more sensitive [43].

Kwon et al. [35] evaluated 97 consecutive dual taper modular stem THAs in a retrospective study by MARS-MRI and stratified 83 of these patients in pseudo-tumor absent (n = 53) and pseudo-tumor present (n = 30). In the pseudo-tumor present group they found no substantial difference between symptomatic (n = 21) and asymptomatic patients (n = 9) in the serum cobalt level (sympt.: 7.6 µg/L (range 3.3–14.4) versus asympt. 6.2 µg/L (range 3.4–11.7)) or cobalt/chromium ratio (sympt.: 8.25 (range 4.5–68) versus asympt. 10.6 (range 4.8–29.5)). For the THA patient group with a pseudo-tumor the cobalt serum level and cobalt/chromium ratio (8.0 µg/L; 10.3) were significantly higher than for those without a pseudo-tumor (2.0 µg/L; 2.4) and based on this the authors suggest cross-sectional imaging (MARS-MRI) for THA patients with elevated metal ion serum levels [35]. In a nano-analysis of wear particles from retrieved peri-prosthetic tissue, Xia et al. [60] could clearly distinguish between metal-on-metal (MoM) surface replacement (n = 12; implantation time 51.6 months), MoM large head THA (n = 18; implantation time 59.9 months) and non-MoM dual modular neck hip arthroplasty (Rejuvenate) (n = 23; implantation time 30.9 months). They found that the particle physical characteristics and metal composition are consistent in each implant category and concluded that substantial differences in size, shape and element composition of the metallic particles correlate with the histological features of severity of ALTR and variability in implant performance, indicating that the immunogenicity and toxicity of the released particles is a leading factor in the specific onset and severity of the reaction [60,61].

## 4. Clinical Case Presentations and Retrieval Analysis

### 4.1. Metha® Dual Taper CoCr/Ti Alloy Couplings with Adverse Local Tissue Reactions

**Figure 1:**
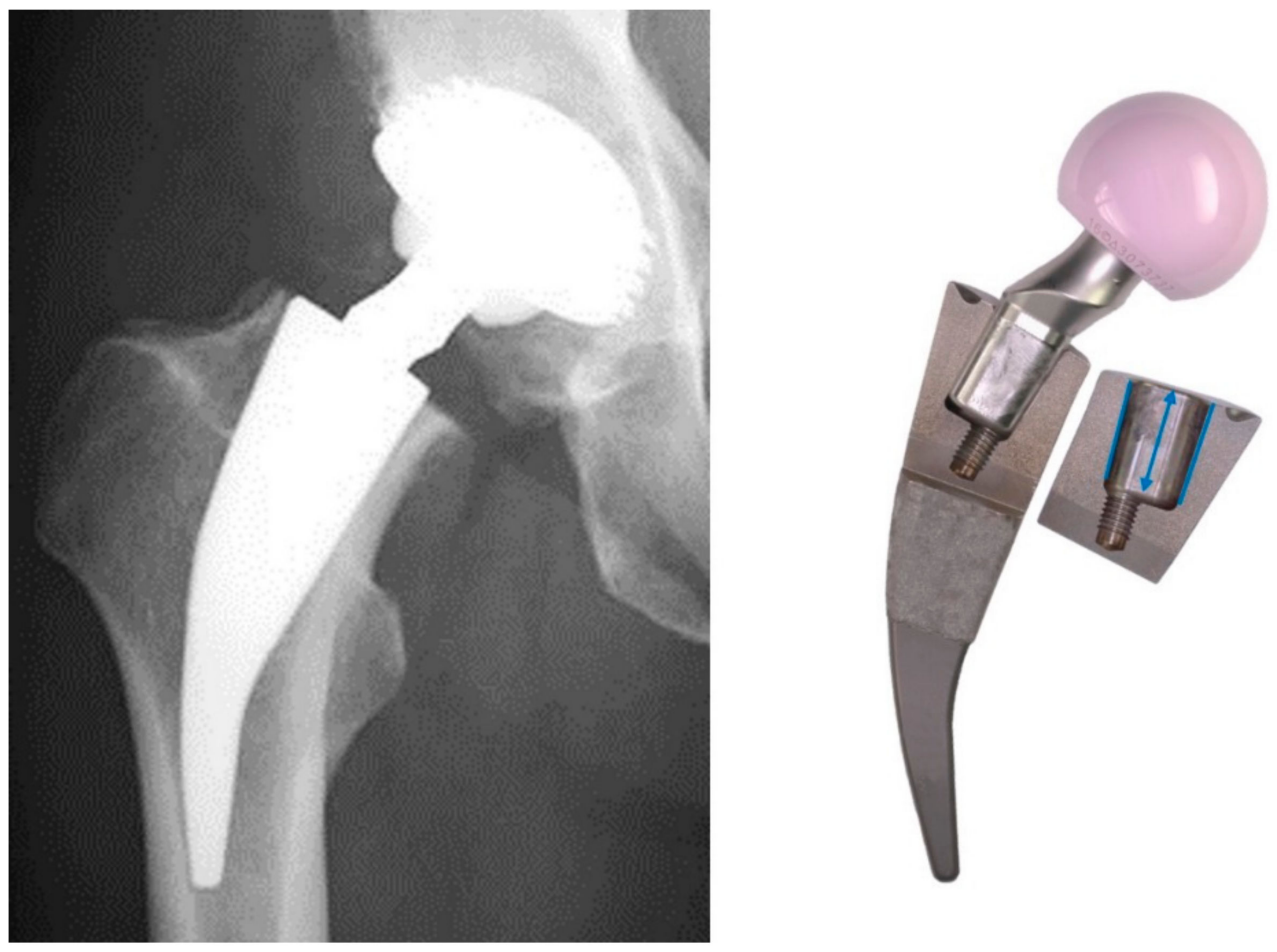
Modular Ti alloy short hip stem with a dual taper CoCr neck—Metha® modular.

Data on all known cases (n = 5) with soft tissue reactions due to debris and metal ion release following THA with the dual modular short stem hip prosthesis Metha® (Aesculap AG, Tuttlingen, Germany; Figure 1) with CoCr neck adapter in the period 2007 to 2019 (Table 2). In single cases (#3 & #5) microbiology and histological analysis was performed and showed a wear particle induced peri-prosthetic interface membrane type I according to the classification of Krenn and Morawietz [62,63]. Average duration between index procedure and revision was 92.6 months (61–128 months).

**Figure 2:**
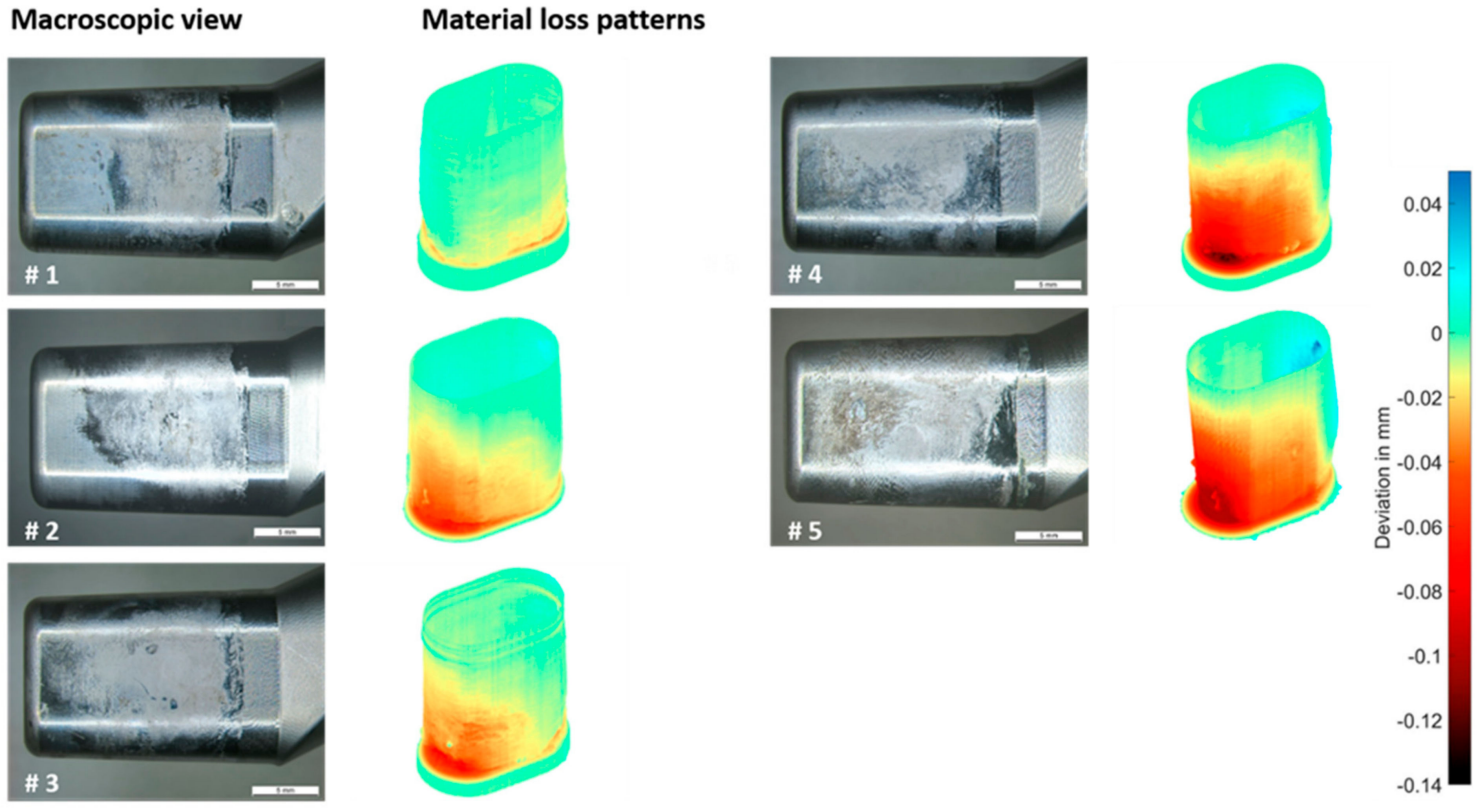
Macroscopic view (left) and volumetric material loss (right) of 5 symptomatic CoCr dual taper neck adapter retrieved after 61–128 months in vivo. The absence of oxygen in the energy dispersive X-ray measurements on characteristic taper surface points indicated that electrochemical processes due to contact, crevice and fretting corrosion affected the ability of the CoCr alloy to form a passive oxide layer in the physiological environment, resulting in increased material loss and metal ion release.

**Figure 3:**
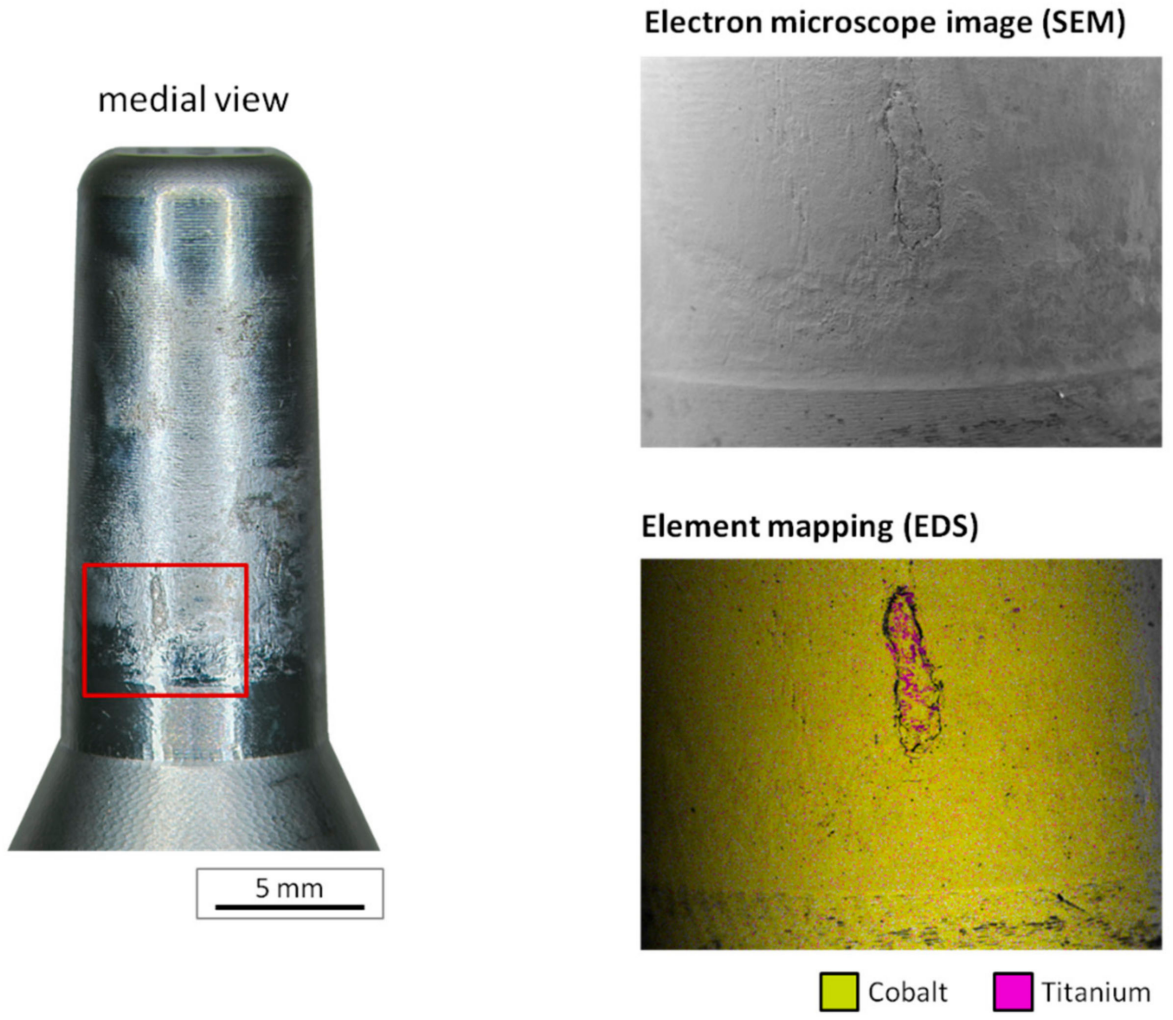
SEM and EDS analysis of the medial contact area of one symptomatic retrieved CoCr neck adapter (Case #3). The SEM and EDS-analysis indicated that the local damage of the protective oxide layer and the suppressed repassivation process led to fretting wear and contact corrosion with metal dissolution in the medio-proximally and latero-proximally contact area of the retrieved neck adapter. Furthermore, local material deposition on the neck adapter with a high element concentration of titanium was observed at the circular medial taper interface caused by local adhesion and cold welding in the mixed CoCr/Ti neck-stem junction.

**Figure 4:**
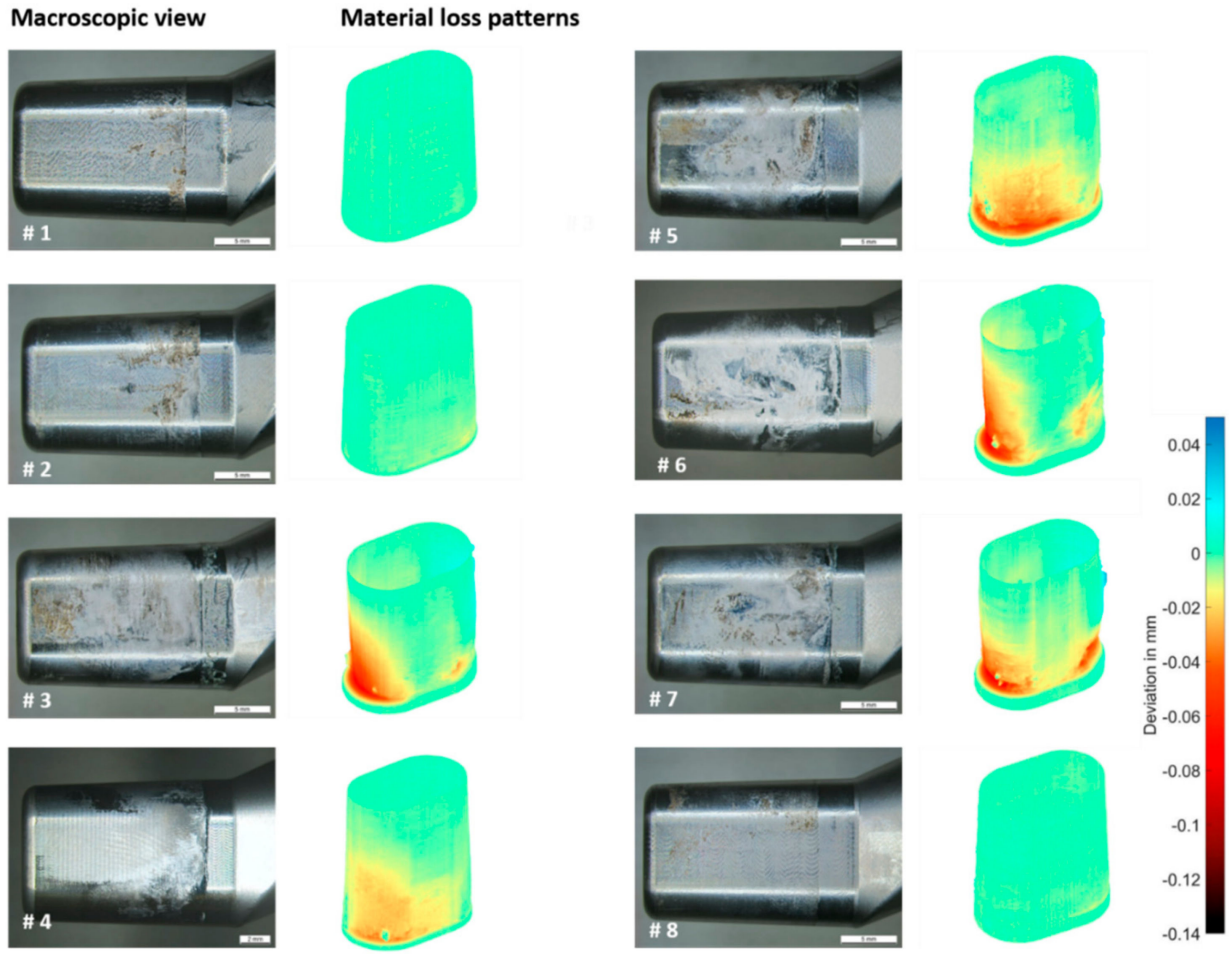
Macroscopic view (left) and volumetric material loss (right) of eight asymptomatic CoCr dual taper neck adapter retrieved after 13–111 months in vivo..

All retrieved CoCr neck components were characterized by means of light microscopy (M165c, Leica Microsystems GmbH, Wetzlar, Germany), scanning electron microscopy (SEM, EVO 50, Carl Zeiss Microscopy GmbH, Jena, Germany) and energy dispersive X-ray analysis (EDS, X-Max 50, Oxford Instruments plc, Abingdon, UK). Volumetric material loss of the neck adapters was quantified according to a previously published algorithm by Buente et al. [64] using a tactile co-ordinate measurement machine (Figure 2, Figure 3 and Figure 4).

Statistics were performed with SPSS 24 (IBM Inc., Armonk, NY USA). Normality was checked using Shapiro-Wilk test. The homogeneity of variances between the groups was checked using Levene’s test. Univariate analysis of variance (ANOVA) was performed to compare the two cohorts linear regression analysis to investigate the dependency of material loss with time in situ. The type 1 error level was set to 5%.

Case 1 (exemplified):

In March 2011, a fifty-one years old woman underwent an uncomplicated total hip arthroplasty of the right hip and received a Metha® (Ti_6_Al_4_V, stem size 2) with a CoCr neck adapter (CCD-angle of 135°, 0° neutral version) and a 40 mm ceramic-on-ceramic articulation with a titanium sleeve (DeltaMotion^®^, Finsbury Orthopaedics, Leatherhead, UK). At 61 months after this index procedure, she presented with progressive pain and swelling localized to the right proximal thigh. The clinical and radiographical evaluation showed no pathologic findings with regard to the range of motion, component position and osseointegration. Magnetic resonance imaging (MRI) demonstrated a large heterogeneous fluid collection (7 cm × 3 cm × 3.4 cm) in the ventral aspect of the iliopsoas muscle. Serum cobalt ion level was elevated at 31.3 µg/L and chromium level was normal at 0.3 µg/L. The positive MRI findings and the high Co/Cr-ratio (ratio = 104) were in keeping with adverse local tissue reactions and pseudo-tumor formation and revision total hip arthroplasty was performed in April 2016. The retrieved modular neck exhibited local surface changes and black deposits on the taper interface. Material analysis showed characteristic fretting and corrosion wear patterns concentrated on the medial and lateral contact region of the neck adapter (Figure 2, neck adapter #1). The largest amount of material loss was observed proximal at the medial taper interface with a maximum wear depth of 37 µm. The total volumetric material loss of the neck adapter was 2.6 mm^3^ (see also Table 2 cases #2–5 and Figure 2, symptomatic neck adapter #2–5).

### 4.2. Retrieval Analysis of CoCr Neck Adapters Revised for Other Reasons than Adverse Local Tissue Reactions

A total of eight asymptomatic Metha® modular CoCr neck adapters revised in the period of 2007 to 2019 for other reasons than adverse local tissue reactions, listed in our database (Table 3). 

The reasons for revision were insufficient osseointegration with migration, cup malpositioning, patient discomfort, cup loosening, luxation, acetabular fracture and periprosthetic fracture. The mean age at time of revision was 68.7 years (63 to 77 years) and the mean period of implantation was 62 months (13 to 111 months). The time to revision for the asymptomatic group is comparably shorter than for the symptomatic group (92.6 months).

Visual inspection of the modular neck adapters of the asymptomatic group (Figure 4) by light microscopy and SEM/ EDS-analysis, demonstrated the same characteristic signs of corrosion and debris concentrated on the medial and lateral surface of the neck/stem interface, but qualitatively less pronounced than for the symptomatic group (Figure 2). One patient (Table 3, #4) with signs of a cup loosening revised after 54 months in situ showed some local tissue reactions, which, however, were classified as asymptomatic due to the low neck adapter wear and material loss (3.0 mm^3^) (Figure 4, neck adapter #4).

Only minor traces of wear and corrosion were seen at the neck adapter of patient #8 (Table 3), based on 111 months of implant in service. The maximum wear depth was 14 µm observed at the proximal lateral taper interface, resulting in a very low total volumetric material loss of 0.3 mm^3^ (Figure 4, neck adapter #8). This may be related to parameters like a low demanding biomechanical loading of the hip, low tribo-corrosion due to a less aggressive joint fluid composition or unknown co-diseases or morbidity of the patient. The adapter was classified as an outlier and not included into the analysis.

**Figure 5:**
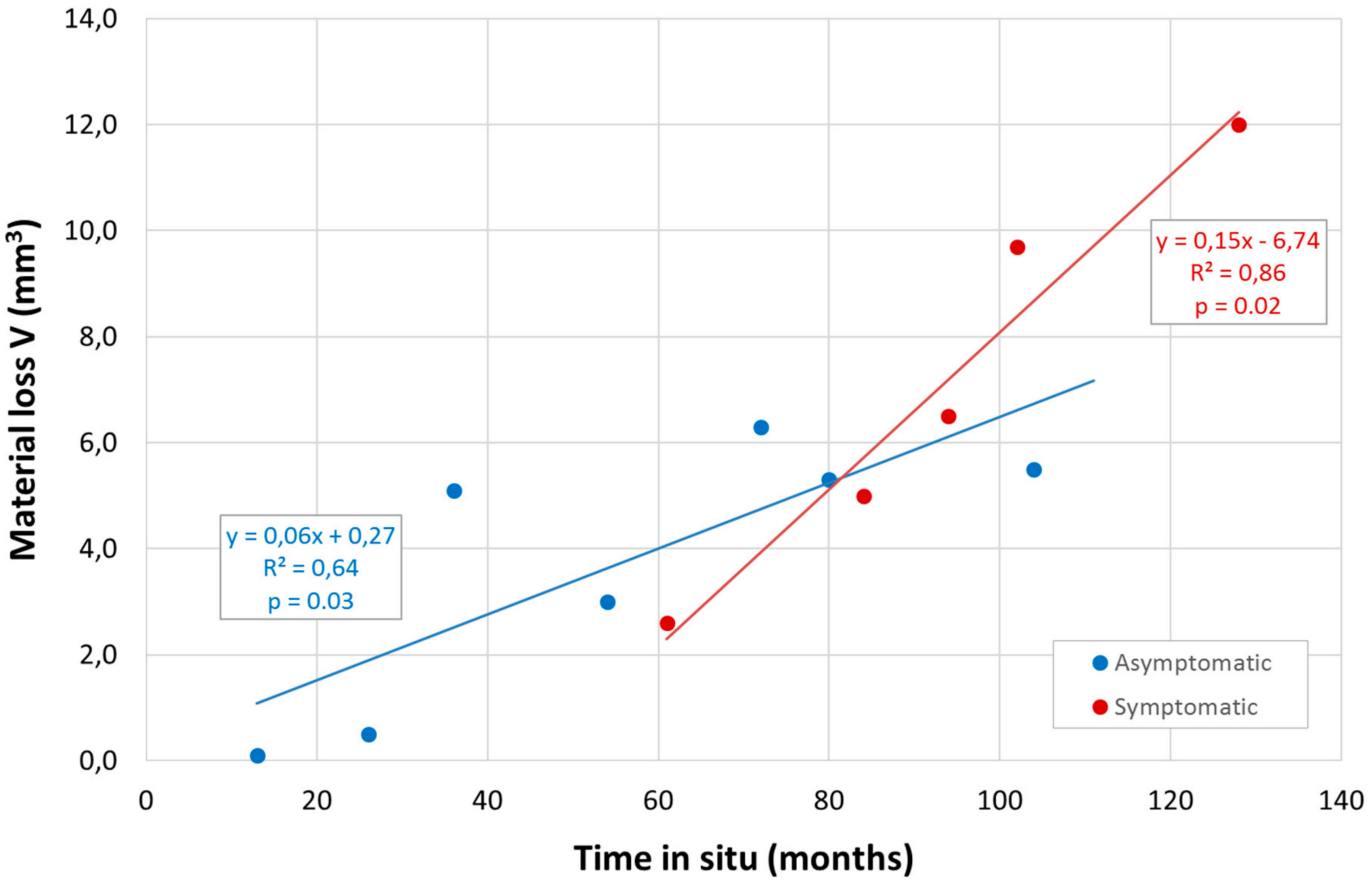
The volumetric material loss versus time in situ of seven asymptomatic and five symptomatic retrieved modular CoCr neck components. Note: asymptomatic neck adapter #8 was excluded from the regression analysis.

The volumetric material loss of the CoCr neck adapters increased for both cohorts with time in situ (Figure 5; adjusted r^2^ = 0.73, p < 0.001). A strong trend for a lower wear rate for the asymptomatic cohort compared to the symptomatic cohort was observed (p = 0.059). The total volume loss for the symptomatic group was 7.16 ± 3.73 mm^3^ and for the asymptomatic 3.69 ± 2.52 mm^3^ (p = 0.082). Time in situ tended to be longer for the symptomatic group (92.6 ± 24.2 months) compared to the asymptomatic group (55.0 ± 32.4 months) (p = 0.054). The two cohorts did not differ with respect to gender distribution or any other patient or implant specific variable shown in Table 2 and Table 3 (p > 0.1 for all analysis).

Due to multiple factors of influence like the patient specific loading situation, weight, activity level, implant orientation and muscular situation, as well as varying physiological surrounding lubricant conditions the comparably low number of only thirteen neck adapters does not allow to estimate a specific patient profile or phenotype. In a descriptive manner of the symptomatic and asymptomatic cases the patient age at revision was 57 to 79 years, their were 12 females and one male, BMI was between 20.2 and 32 (unknown in 6 cases), neck adapter CCD angles were 130° and 135°, and the head material was ceramic in all cases (2/13 ceramic heads with Ti-Sleeve).

## 5. Decision Making Model for Asymptomatic Dual Taper Stems in Case of Acetabular Revision

**Scheme 2:**
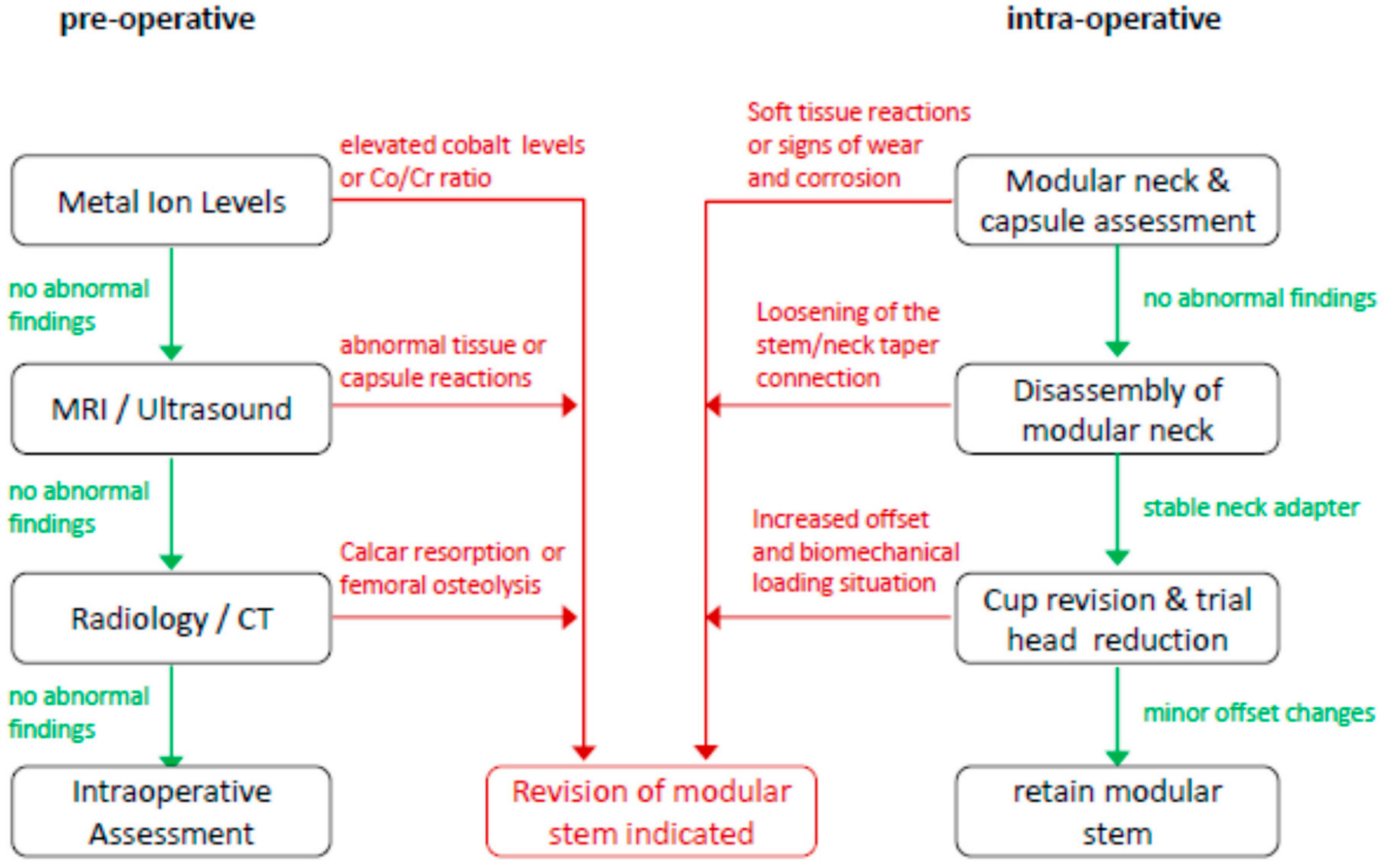
Practical guide how to proceed with an asymptomatic dual taper modular hip stem in case of acetabular revision.

In the present study, metal ion concentrations were not determined. Therefore the available data from the literature were utilized to develop a decision making model for the case of acetabular revision for pre- and intra-operative decision making, as an orientation how to decide whether to maintain or revise an asymptomatic dual taper stem (Scheme 2).

Cobalt and chromium ions are released from modular dual taper stem connections as consequence of mechanically assisted crevice corrosion [17]. In several retrieval studies elevated cobalt ion levels were associated with adverse local tissue reactions in THA patients with dual taper stems [18,39,45] and a cobalt value of ≥ 8 µg/L serum concentration has been documented for patients having a symptomatic dual taper stem and or a pseudo-tumor present [31,35,39,43,44,49,65,66]. An additional important diagnostic parameter is differential elevation of cobalt relative to chromium [58,65], originated by a predominant cobalt ion release at modular taper connections related to a chemical corrosion process that involves more soluble cobalt dissipating as free ions [40,45,67].

In a series of 447 consecutive patients tested for serum levels Fillingham et al. [65] identified 64 patients with a metal-on-polyethylene THA bearing (12 with a dual taper modular neck), whereas 44 were positive for an adverse local tissue reaction. The diagnosic measures showed a mean serum cobalt level of 8.58 µg/L and a Co/Cr ratio of 11.56. Kwon et al. [35] performed a retrospective study of 97 consecutive patients with a dual taper femoral stem and found substantially elevated cobalt levels of 8.0 µg/L (3.3–14.4) and an elevated Co/Cr ratio of 10.3 (4.5–68.0) in their pseudo-tumor present group. Ghanem et al. [43] identified 107 patients who underwent 118 THAs (11 bilateral cases) with a Rejuvenate dual taper femoral stem and proposed a decision tree to detect whether or not symptoms were present. They found that patients with a serum cobalt level < 6.25 µg/L had a chance of 82% to stay without symptoms, while those with ≥ 18.5 µg/L had a very high risk of failure. Patients in the failure group had a mean cobalt concentration of 9.5 µg/L and a mean Co/Cr ratio of 5.2, whereas the asymptomatic group had a concentration of 4.9 µg/L and a ratio of 3.6.

In a recent study 148 patients with dual taper modular THA (110 Rejuvenate, 38 ABG II) were examined for pseudo-tumors (n = 90) on MARS-MRI and the severity of intra-operative tissue reactions was correlated with pre-operative cobalt ion levels [36]. The occurrence of pseudo-tumors was associated with significantly elevated cobalt levels (5.0 mg/L vs 3.7 mg/L), a higher Co/Cr ratio (6.0 vs 3.7) and also higher intra-operative tissue damage grades demonstrated substantially elevated Co/Cr ratios (8.6 vs 3.4). These findings on cobalt values and Co/Cr ratios were also underligned by a comparison of cobalt and chromium level diagnostic measurements between positive and negative ALTR groups for a specific metal-on-polyethylene head-neck modularity THA design without dual-taper neck (cohort n = 62) [68]. In 43 THA patients with ALTR a mean cobalt level of 8.92 µg/L and a Co/Cr ratio of 5.91 were found.

In a consensus statement of the American Association of Hip and Knee Surgeons, the American Academy of Orthopaedic Surgeons and the Hip Society, Kwon et al. [58] defined a “high risk” group stratification combining a serum cobalt level > 5 µg/L and a Co/Cr ratio > 5 as factors for diverse modular taper junctions associated with adverse local tissue reactions.

Investigating a cohort of 123 Rejuvenate dual taper THAs, Meftah et al. [31] described a comparably high revision free probability for patients with cobalt serum levels < 4 µg/L. In addition their patients with a Co/Cr ratio of < 3.6 had a likelihood to stay within the asymptomatic group as it has been similarly analysed by Ghanem et al. [43] and Kwon et al. [36].

Due to the fact that serum cobalt ion levels and Co/Cr ratio are confounded in patients having a contra-lateral head-neck-trunnionosis, a bilateral dual taper stem, a metal-on-metal bearing or another joint replacement (knee, shoulder, ankle), surgeons should not solely rely on ion serum concentration factors to determine a clinical recommendation for stem revision [58,65].

An aspiration of the hip to rule out peri-prosthetic infection [69] and to perform intra-articular synovial fluid collection for cobalt and chromium ion content analyses may be a considerable diagnostic option to detect symptomatic MACC prior acetabular revision THA [48,70]. McGrory et al. [70] examined the relationship between serum and intra-articular (IA) cobalt and chromium levels in a cohort of 16 patients with symptomatic MACC undergoing hip revision and they concluded that intra-articular joint fluid levels (IA cobalt 940 µg/L; IA chromium 491 µg/L) positively correlated with serum levels (cobalt 5.1 µg/L; chromium 1.3 µg/L), but intra-articular levels were on average 100-fold higher.

As an important diagnostic tool in detection of adverse tissue reactions due to dual-taper fretting wear and corrosion, cross sectional imaging modality by metal artifact reduction sequence magnetic resonance imaging (MARS-MRI) [35,48,58,59,71,72,73,74,75] and musculoskeletal ultrasound (US) [76] have been qualified.

Walsh et al. [75] described the incidence of different pathologies based on MARS-MRI images in a retrospective cohort of 312 THAs in 272 patients with a dual taper CoCr neck and a beta-titanium alloy stem implanted between 2007 and 2012. They noted synovitis in 167 hips (53.5%), osteolysis in 18 hips (5.8%) and an effusion or fluid collection in 194 hips (62.3%), whereas 52 (29.1%) of these fluid collections were noted to contain debris. Solely intra-capsular effusion and fluid collection was found in 127 hips (40.7%) and combined intra- and extra-capsular in 52 hips (16.7%) [48]. Tendinopathy of one of the related muscle groups (glutaeus minimus, glutaeus medius, iliopsoas or hamstrings) was seen in 250 (80.1%) of the hips and in 87 (27.9%) some tendon disruption occurred.

The presence of a thickened capsule in association with an effusion is a common MARS-MRI abnormality often accompanied by findings like iliopsoas and abductor tendinopathy, peri-tendinous collections and also the presence of metallic debris [59]. For the detection of adverse local tissue reactions like solid or cystic pseudo-tumors MARS-MRI is a highly sensitive modality [59].

Barlow et al. [71] performed in a revised cohort of 90 THA patients with 98 Rejuvenate modular femoral neck stems MRI and serum cobalt and chromium ion level analysis before revision and used histologic samples from revision surgery to score for synovial lining, inflammatory infiltrate and tissue organization according to Campbell et al. [77]. They found that MRI enables to accurately describe ALTR in dual taper modular neck THA patients and they predicted histologic severity particularly based on maximal synovial thickness and synovitis volume.

To identify femoral osteolysis, loosening and erosions in trochanteric or calcar regions possibly associated with taper corrosion, a focused review of a series of plain antero-posterior and lateral radiographs is proposed [59,69].

Werner et al. [78] reported about adverse inflammatory soft tissue reactions as a consequence of enhanced wear and corrosion of a dual taper neck-stem interface. At time of revision they describe extensive debridement of pseudo-tumor and necrotic bursal and capsular tissue encapsulating the hip joint, as well as corrosion at the neck-stem interface with significant black corrosive debris throughout the modular neck and the soft tissues [78]. Walsh et al. [48] published a study on a cohort of 99 patients including 103 revisions of a dual taper modular neck stem (78 Rejuvenate, 25 ABG II) at a mean time of 2.4 years from primary surgery. They reported intra-operative findings of the 103 revised hips, observing a black metallic sludge associated from corrosion and wear debris in all hips (100%), bony calcar erosion in 88/103 (85.4%), pseudo-tumor formation in 26/103 (25.2%), peri-capsular necrosis in 84/103 (81.6%), tissue necrosis in 80/103 (77.7%) and synovitis in 101/103 (98.1%) of the cases.

Dimitriou et al. [33] evaluated 198 revision surgeries of a dual taper modular femoral stem in 187 THA patients, by an intra-operative tissue damage grading system and observed adverse tissue reactions in 178 (89%), a large amount of fluid entering the capsule in 160 (81%) and particulate wear debris in 103 (52%) of the hips, whereby in all cases a black metallic corrosion debris was found.

If a disassembling of the modular neck takes place intra-operatively during removal of the femoral head within an acetabular revision procedure, the modular hip stem shall be considered for revision. The reason for this is, that a not firmly fixed modular neck/stem taper connection is of high risk for mechanically assisted crevice corrosion. A loosened neck/stem taper connection can be originated during index surgery by an insufficient assembling force or can be caused by macroscopic visible material loss at the medial neck taper interface due to corrosion resulting in a toggling of the modular CoCr neck relatively to the Ti alloy stem [79]. 

During acetabular cup revision, it may be necessary to place the new cup in an anatomically different position compared to index surgery and this may impact the necessary offset and neck length of the femoral head. In addition, the restoration of the centre of rotation and related ligament balancing during trial head reduction possibly requires a higher offset or longer neck length [4,7,80,81]. Increasing the offset or neck length to adapt for a different cup orientation or sufficient soft tissue balancing may create a more demanding biomechanical loading situation [7,16,32,82,83,84] for the so far asymptomatic dual taper neck adapter, possibly triggering MACC [17,32,79]. Therefore an indication may be given for revision of the modular stem.

## 6. Discussion

In an attempt to find an answer to the question—How to proceed with a clinically asymptomatic modular Metha® stem with dual taper CoCr neck adapter in case of acetabular revision?—following systematic methods have been applied:

1) The status of research and appropriate diagnostic methods in context to clinically symptomatic and asymptomatic dual taper stem-neck couplings was evaluated based on a systematic literature review.
2) A retrieval analysis of thirteen Metha® dual taper CoCr/ Ti alloy hip stems was performed.
3) A rational decision making model as basis for a clinical recommendation was developed.

A limitation may arise by the fact that the literature review about serum ion levels, radiographic and clinical findings and the retrieval analysis was based in the vast majority on two recalled dual taper stems in the material combination CoCr_29_Mo_6_ (neck) and TiMo_12_Zr_6_Fe_2_ (stem), and hence a generalization of the findings and transfer to other implant designs and materials of dual taper stems may be limited [64,85,86]. Meftah et al. [31] used persistent pain and high cobalt levels as predictors for revision surgery of the dual taper Rejuvenate stem and reported a Kaplan-Meier survivorship of 40% at four years with revision related to neck taper corrosion as the end point. They calculated a revision-free probability of 93% for patients with cobalt levels of less than 4.0 µg/L compared with 45% for those with cobalt levels of 4.0 µg/L and found that significantly higher metal ion levels correlated with younger age and a higher femoral offset [31]. Bernstein et al. [44] described a corrosion-related revision rate of 28% at a mean follow-up of 2.7 years in a cohort of 81 Rejuvenate modular hip stems. They prospectively followed this cohort of patients with elevated serum cobalt ion levels (> 4 µg/L), persistent pain, or abnormal MRI findings as indications for revision and observed a clinical failure rate of 86% at a mean of 4.2 years [44]. Koziara et al. [87] reported a study group of 66 out of a cohort of 156 patients who underwent modular Rejuvenate THA with an average follow-up of 55 months (range 22–89) with a revision rate of 31.8% (21 of 66 THAs). They found in the non-revision group a mean serum cobalt ion level of 3.48 µg/L and a Visual Analog Scale (VAS) pain score of 2.4, whereas for the revision group a level of Co = 5.05 µg/L and a VAS pain score 5.1 was present. From the revised group, 18 patients were undergoing MARS-MRI. The THA patients who did not have reactive tissue showed a mean serum cobalt ion level of 4.15 µg/L and a VAS pain score 3.8 and in the group with reactive tissue formation cobalt ion level was Co = 5.01 µg/L and a VAS pain score of 5.63 [87].

For a cohort of 36 patients who underwent uni-lateral primary THA with Profemur® Preserve Ti_6_Al_4_V alloy femoral stems and ceramic-on-ceramic bearings, Barry et al. [88] determined the impact of the modular neck material Ti alloy (n = 22) or CoCr (n = 14) with no patient being revised. With a comparably short-term follow-up of 20 months (range 9–44), they observed significantly higher cobalt ion serum concentrations in the CoCr neck group (0.46 vs 0.26 µg/L) and higher titanium ion serum concentrations in the Ti alloy neck group (1.98 vs 1.59 µg/L), but on a comparably low level. Laurencon et al. [89] reported serum and whole blood metal ion levels of a prospective cohort study on 40 THA patients with a cementless anatomic SPS stem made of Ti_6_Al_4_V alloy with modular CoCr necks with a mean follow-up of 23 months (range 12–28) and found in 6 of 40 (15%) serum cobalt ion levels > 2 µg/L and in 3 of 40 (7.5%) values > 4 µg/L. Applying MARS-MRI in all THA patients with a serum ion level > 2 µg/L, they detected a pseudo-tumor in one patient having a serum level of 5.21 µg/L for cobalt, 3.51 for chrome and 42 µg/L for titanium [89]. Using a nationwide retrospective cohort of 324,108 THA patients from the French health insurance system, Colas et al. [90] described a cumulative revision incidence of 6.5% for exchangeable neck THAs (n = 8,931) versus 4.7% for fixed neck THAs (n = 315,177) and a significantly increased adjusted hazard ratio of revision of 1.26.

In the National Joint Replacement Registry Report 2018 of the Australian Orthopaedic Association for exchangeable femoral neck adapters the cumulative percent revision for primary THA was reported to be 4.9% at 5 years and 6.8% at 10 years for Ti alloy-Ti alloy stem-neck couplings [91]. For Ti alloy-CoCr modular neck couplings they reported a cumulative percent revision of 9.6% at 5 years and of 16.6% at 10 years [91].

In THA femoral stems with modular exchangeable neck components had significantly lower 10-year survival rates in literature reviews and in registry data compared to primary THA implant survivorship for femoral mono-bloc stems [92,93].

Su et al. [94] performed a retrieval analysis for neck fretting and corrosion on 60 Rejuvenate modular stem designs and compared those to 26 retrieved implants from seven other modular CoCr and Ti alloy neck designs. For the Rejuvenate design they found significantly higher damage and corrosion scores and a 20-fold increased likelihood to show ALVAL based on histologic samples, than for the other designs. As a relevant parameter they stated—beyond design aspects—the lower Youngs modulus of 80 GPa for the TiMo_12_Zr_6_Fe_2_ stem material (Ti_6_Al_4_V; 110 GPa), being responsible for increased metal transfer and surface damage in coupling with a CoCr neck, which could account for the higher ALVAL and corrosion scores [94].

Somers et al. [95] stated for the market withdrawn Rejuvenate and ABG II hip systems that the different design features and the stem material TiMo_12_Zr_6_Fe_2_ show more fretting corrosion and it is possible that the different metal trace elements (molybdenum, zirconium and iron) might lead to a more pronounced toxic local tissue reaction.

Lewinski and Floerkemeier [13] described their 10-year experience with short stem TH based on 1953 Metha® short stem procedures with an overall aseptic stem revision rate of 1.3% and 1.9% including 12 modular Ti alloy neck adapter failures based on 190 modular stems with Ti alloy necks implanted before the product recall in November 2006 [16]. Schnurr et al. [14] recorded data for 1888 Metha® short stem implantations from 2004 to 2014 with three implanted versions: Modular Ti_6_Al_4_V alloy stems with Ti alloy (n = 314) or CoCr (n = 230) neck adapters and mono-bloc Ti alloy stems (n = 1090) with a mean follow-up of 6 years (range 1–11). They found a 7-year revision rate for mono-bloc of 1.5%, for modular cobalt-chrome of 1.8% and for modular Ti alloy adapter stems of 5.3%, including 15 modular Ti alloy neck fractures.

Apart of the promising 7 to 10 years clinical experiences with the modular Metha® short stem with CoCr necks [13,14], a limitation of our retrieval analysis study is the small number of symptomatic (n = 5) and asymptomatic (n = 8) retrieved CoCr neck adapters out of a cohort of 25,177 dual taper modular stems with CoCr neck, implanted from January 2007 until January 2017. On the other hand side compared to the mostly short-term follow-up in the literature [31,43,44,70,87,88,89] the retrievals in our study have an average follow-up of 73.8 months and 6 of them have been in patients service for more than 7 years (range 84–128 months).

## 7. Conclusions

Based on the analysis of the literature it is suggested that in case of required acetabular revision in patients with a serum cobalt level of > 4 µg/L [31,33,35,36,43,44,58,87] and a Co/Cr ratio > 3.6 [33,35,36,42,43,58] revision of the modular dual taper stem may be considered.

Prior acetabular revision surgery in patients with dual taper modular neck stem THA [59], a systematic diagnostic evaluation has to be executed, using specific tests such as serum metal (Co, Cr) ion analysis, plain antero-posterior and lateral radiographs [59,69] and cross-sectional imaging modalities (MARS-MRI, US) [48,55,59,77]. The patient’s stated pain level (e.g., VAS pain score) should also be included as an important factor and measurements of IA cobalt and chromium levels may be meaningful [70].

For an asymptomatic Metha® dual taper Ti alloy/CoCr stem-neck coupling at stage of acetabular revision, careful clinical decision making according to the proposed model should be followed and overreliance on any single examination should be avoided, considering the complete individual differential diagnosis and patient situation.

## Figures and Tables

**Table 1:** Literature overview about clinical cohorts with dual taper neck adapter.

Study	Prosthesis	Neck/ Stem Material	Mean Age (Years)	Follow-up (Years)	Number of Patients	Study Group	Serum Level (µg/L)			Co/Cr Ratio	MRI Findings
							Co	Cr	Ti		
Omlor et al. 2013 [37]	Profemur E	Ti6Al4V/Ti6Al4V	66.0	9.0	67	Modular (Uni-/bilateral)	-	-	3.0/6.0	-	-
	CLS	Ti6Al4V	71.0		11	Non-modular (Uni-/bilateral)	-	-	2.7/6.2	-	-
Molloy et al. 2014 [42]	ABGII	Vitallium/TMZF	64.3	3.4 3.6	7 8	Revised Non-revised	6.3 3.9	1.3 1.1	- -	6.4 3.7	86% 13%
Silverton et al. 2014 [50]	Profemur Z	Ti6Al4V/Ti6Al4V	59.5	4.5	137	Follow-up	2.4	1.7	4.2	-	-
Gofton et al. 2015 [38]	Profemur TL	Ti6Al4V/Ti6Al4V	60.2	2.0	25 25	MoM articulation MoP articulation	2.5 0.3	2.1 0.3	2.8 2.9	- -	- -
Lanting et al. 2015 [51]	Rejuvenate	Vitallium/TMZF	65.0	1.7	19	Revised	5.5	0.8	3.0	8.2	83%
Restrepo et al. 2014 [52]	ABGII	Vitallium/TMZF	61.0	2.0	85 110	Symptomatic Asymptomatic	4.0 3.4	1.2 1.2	- -		46% 11%
Barlow et al. 2016 [55]	Rejuvenate	Vitallium/TMZF	65.8	2.4	90	Revised	6.6	1.3	-	-	81%
Chillemi et al. 2017 [53]	ABGII	Vitallium/TMZF	69.4 65.1	>1.0	5 17	Symptomatic Asymptomatic	3.0 3.6	0.4 0.7	- -	7.4 9.6	- -
Kwon et al. 2017 [36]	Rejuvenate/ABGII	Vitallium/TMZF	59.0	1.4	90	Pseudotumor	5.0	0.8	-	6.0	100%
			58.0	1.3	58	No pseudotumor	3.7	0.8	-	3.7	0 %
Liow et al. 2016 [54]	Rejuvenate/ABGII	Vitallium/TMZF	57.4	2.3	31	Revised	3.8	1.0	-	3.8	100 %

**Table 2:** Overview of five symptomatic clinical cases with retrieved Metha^®^ CoCr dual taper neck adapter.

Patient (n)	Indication & Revision Procedure	Time In Situ (mths)	Sex	Age (years)	BMI (kg/m^2^)	Stem Size & Side	CCD-Angle Version	Head Size & Offset	Head Material	Serum Level (µg/L)	Co/Cr Ratio	Max. Wear Depth (µm)	Material Loss (mm^3^)
1	Soft tissue reactions progressive pain & swelling, ALTR & pseudo-tumor formation	61	Female	60	-	2 right	135° 0°	40 +0	Ceramic/Ti-Sleeve	Co=31.3 Cr=0.3	R=104	37	2.6
2	Groin pain, hip aspiration—cloudy-yellow synovial fluid, local tissue infiltrations, thickened capsular tissue	84	Female	67	-	3 left	135° 0°	36 −4.0	Ceramic	-	-	72	5.0
3	Soft tissue reactions, persistent hip pain ALTR, positive MRI findings, peri-prosthetic joint infection, extensive debridement of capsular tissue & metal debris	94	Female	76	32.0	2 left	130° 7.5° AV	32 +4.0	Ceramic	Co=8.3 Cr=0.8	R=10.37	82	6.5
4	Soft tissue reactions, persistent hip pain & swelling, elevated Co level, pseudo-tumor resection & debridement of metal debris, severe loosening of ScrewCup^®^ SC	96	Female	58	24.6	2 -	135° 0°	32 −4.0	Ceramic	-	-	128	9.7
5	CT hip scan, migrated Hofer cup, suspicion cup loosening, exploration oft he hip, accumulation of metal debris, muddy-yellow fluid accumulation within joint capsula	128	Female	79	-	2 left	135° 7.5°AV	36 +0	Ceramic	-	-	100	12.0

**Table 3:** Overview of eight asymptomatic clinical cases with retrieved CoCr dual taper neck adapter.

Patient (n)	Revision	Time In Situ (mths)	Sex	Age (Years)	BMI (kg/m^2^)	Stem Size & Side	CCD-Angle Version	Head Size (mm)	Head Offset (mm)	Head Material	Max. Wear Depth (µm)	Material Loss (mm^3^)
1	Osseointegration insufficient	13	Male	73	29.7	5 right	130° 0°	36	+4.0	Ceramic	7	0.1
2	Malposition cup	26	Female	77	31.6	1 right	130° 0°	32	−4.0	Ceramic	16	0.5
3	Patient discomfort	36	Female	63	20.2	2 right	135° 0°	32	−4.0	Ceramic	78	5.1
4	Cup loosening	54	Female	69	-	2 left	135° 0°	36	−4.0	Ceramic	39	3.0
5	Patient discomfort	72	Female	67	-	3 right	130° 0°	32		Ceramic/Ti-Sleeve	86	6.3
6	Luxation	80	Female	63	29.1	3 left	130° 0°	32	−4.0	Ceramic	61	5.3
7	Acetabulum fracture	104	Female	63	25.2	2 right	135° 0°	28	+5.0	Ceramic	90	5.5
8	Periprosthetic fracture	111	Female	74	-	3 right	135° 7.5° RV	28	+3.5	Ceramic	14	0.3
